# *TLR7 *single-nucleotide polymorphisms in the 3' untranslated region and intron 2 independently contribute to systemic lupus erythematosus in Japanese women: a case-control association study

**DOI:** 10.1186/ar3277

**Published:** 2011-03-11

**Authors:** Aya Kawasaki, Hiroshi Furukawa, Yuya Kondo, Satoshi Ito, Taichi Hayashi, Makio Kusaoi, Isao Matsumoto, Shigeto Tohma, Yoshinari Takasaki, Hiroshi Hashimoto, Takayuki Sumida, Naoyuki Tsuchiya

**Affiliations:** 1 Molecular and Genetic Epidemiology Laboratory, Doctoral Program in Biomedical Sciences, Graduate School of Comprehensive Human Sciences, University of Tsukuba, 1-1-1 Tennodai, Tsukuba 305-8575, Japan; 2 Department of Rheumatology, Clinical Research Center for Allergy and Rheumatology, Sagamihara National Hospital, National Hospital Organization, 18-1 Sakuradai, Minami-ku, Sagamihara 252-0392, Japan; 3 Division of Clinical Immunology, Doctoral Program in Clinical Sciences, Graduate School of Comprehensive Human Sciences, University of Tsukuba, 1-1-1 Tennodai, Tsukuba 305-8575, Japan; 4 Department of Rheumatology, Niigata Rheumatic Center, 1-2-8 Hon-cho, Shibata 957-0054, Japan; 5 Division of Rheumatology, Department of Internal Medicine, Juntendo University, 2-1-1 Hongo, Bunkyo-ku, Tokyo 113-8421, Japan; 6 Juntendo University School of Medicine, 2-1-1 Hongo, Bunkyo-ku, Tokyo 113-8421, Japan

## Abstract

**Introduction:**

The Toll-like receptor 7 (*TLR7*) gene, encoded on human chromosome Xp22.3, is crucial for type I interferon production. A recent multicenter study in East Asian populations, comprising Chinese, Korean and Japanese participants, identified an association of a *TLR7 *single-nucleotide polymorphism (SNP) located in the 3' untranslated region (3' UTR), rs3853839, with systemic lupus erythematosus (SLE), especially in males, although some difference was observed among the tested populations. To test whether additional polymorphisms contribute to SLE in Japanese, we systematically analyzed the association of *TLR7 *with SLE in a Japanese female population.

**Methods:**

A case-control association study was conducted on eight tag SNPs in the *TLR7 *region, including rs3853839, in 344 Japanese females with SLE and 274 healthy female controls.

**Results:**

In addition to rs3853839, two SNPs in intron 2, rs179019 and rs179010, which were in moderate linkage disequilibrium with each other (*r*^*2 *^= 0.53), showed an association with SLE (rs179019: *P *= 0.016, odds ratio (OR) 2.02, 95% confidence interval (95% CI) 1.15 to 3.54; rs179010: *P *= 0.018, OR 1.75, 95% CI 1.10 to 2.80 (both under the recessive model)). Conditional logistic regression analysis revealed that the association of the intronic SNPs and the 3' UTR SNP remained significant after we adjusted them for each other. When only the patients and controls carrying the risk genotypes at the 3' UTR SNPpositionwere analyzed, the risk of SLE was significantly increased when the individuals also carried the risk genotypes at both of the intronic SNPs (*P *= 0.0043, OR 2.45, 95% CI 1.31 to 4.60). Furthermore, the haplotype containing the intronic risk alleles in addition to the 3' UTR risk allele was associated with SLE under the recessive model (*P *= 0.016, OR 2.37, 95% CI 1.17 to 4.80), but other haplotypes were not associated with SLE.

**Conclusions:**

The *TLR7 *intronic SNPs rs179019 and rs179010 are associated with SLE independently of the 3' UTR SNP rs3853839 in Japanese women. Our findings support a role of *TLR7 *in predisposition for SLE in Asian populations.

## Introduction

Toll-like receptors (TLRs) play a central role in detecting microbial pathogens. TLRs initiate innate immune responses and also induce adaptive immune responses [1]. Recently, TLRs have been strongly implicated in autoimmune diseases [2]. The *TLR7 *and *TLR9 *genes, which are expressed intracellularly in plasmacytoid dendritic cells (pDCs) and B cells, recognize single-stranded RNA and DNA containing cytidine-phosphate-guanosine motifs, respectively. Activation of pDCs by TLR7 and TLR9 induces a large amount of type I interferon (IFN). It has become evident that RNA- and DNA-containing immune complexes, which often exist in sera of patients with systemic lupus erythematosus (SLE), can activate TLR7 and TLR9 signaling [2].

Several lines of evidence support a role of TLR7 in SLE pathogenesis [2]. Male BXSB mice bearing the Y chromosome-linked autoimmune accelerator (*Yaa*) gene develop severe SLE. It has been revealed that *Yaa *mutation is caused by a translocation of a portion of the X chromosome containing *TLR7 *onto the Y chromosome [3,4]. *Yaa*-bearing mice have been demonstrated to have twofold overexpression of TLR7 protein and mRNA [3,4]. In contrast, lupus-prone MRL/Mp^*lpr*^^/^^*lpr *^mice lacking *TLR7 *showed impaired production of antibodies to RNA-containing antigens, such as anti-Smith (anti-Sm) antibodies, and developed less severe disease [5]. Furthermore, upregulated expression of *TLR7 *mRNA in peripheral blood mononuclear cells (PBMNCs) was observed in human SLE [6].

Recently, a multicenter collaborative study including our group reported an association of *TLR7*, located in Xp22.3, with SLE in combined East Asian populations [7]. In a discovery panel consisting mainly of Chinese and Korean populations, the association of 27 single-nucleotide polymorphisms (SNPs) in the *TLR7*-*TLR8 *region with SLE was examined, and a significant association of the *TLR7 *3' untranslated region (3' UTR) SNP, rs3853839, was identified. Subsequently, the association of rs3853839 was replicated in two independent Chinese and Japanese case-control sets. The association was prominent in males with SLE. In addition, rs3853839 was associated with elevated expression of *TLR7*. The study also revealed some differences in the association of rs3853839 and other SNPs among Chinese, Korean and Japanese populations [7], indicating that systematic SNP screening should be performed in each population.

In this study, we examined the association of eight *TLR7 *tag SNPs with SLE in Japanese women and discovered a newly identified association of two intronic SNPs, rs179019 and rs179010, with SLE. These SNPs and the 3'UTR rs3853839 were found to independently contribute to the genetic risk for SLE.

## Materials and methods

### Patients and controls

Three hundred forty-four Japanese female patients with SLE (mean age ± SD, 42.9 ± 13.8 years) and 274 healthy female controls (mean age ± SD, 31.3 ± 8.9 years) were recruited at University of Tsukuba, Juntendo University, Sagamihara National Hospital, and at the University of Tokyo. Among them, 296 SLE patients and 250 healthy controls were also examined in a previous study to replicate the association of rs3853839 with SLE in Japanese, but other SNPs were not investigated in that study [7]. All patients and healthy individuals were native Japanese living in the central part of Japan. All patients with SLE fulfilled the American College of Rheumatology criteria for SLE [8].

This study was carried out in compliance with the Declaration of Helsinki. The study was reviewed and approved by the research ethics committees of University of Tsukuba, Sagamihara National Hospital, the University of Tokyo and Juntendo University. Informed consent was obtained from all study participants.

### Genotyping

Eight tag SNPs in the *TLR7 *region were selected on the basis of the HapMap Phase II JPT (Japanese in Tokyo) data obtained from the HapMap database [9] with the criteria of minor allele frequency >0.1 and an *r*^2 ^threshold of 0.9. Genotyping of the tag SNPs was carried out using the TaqMan genotyping assay on the Applied Biosystems 7300 Real-Time PCR System (Applied Biosystems, Foster City, CA, USA), according to the manufacturer's instructions. Thermal cycling conditions consisted of initial denaturation at 95°C for 10 minutes, followed by 50 cycles at 95°C for 15 seconds each and at 60°C for one minute each. TaqMan probes used in this study were as follows: Assay ID: C__15757400_10 (rs2302267), C___2259585_10 (rs179019), C___7625717_10 (rs1634322), C___2259582_10 (rs179016), C___2259578_10 (rs179012), C___2259576_10 (rs179010), C___2259575_10 (rs179009), and C___2259573_10 (rs3853839).

### Expression analysis by real-time quantitative reverse transcription polymerase chain reaction assay

Total RNA was extracted from PBMNCs of 18 females with SLE using the RNeasy Mini Kit (QIAGEN, Hilden, Germany), reverse transcribed into cDNA and used for real-time quantitative reverse transcription polymerase chain reaction (RT-PCR) assay. Expression of *TLR7 *was analyzed using the TaqMan Gene Expression Assay (Applied Biosystems), Hs00152971_m1. Amplification of cDNA was conducted using the Applied Biosystems 7300 Real-Time PCR System (Applied Biosystems) under the following conditions: 50°C for 2 minutes and 95°C for 10 minutes, and 50 cycles at 95°C for 15 seconds and at 60°C for 1 minute, and then the cycle threshold (CT) value for each sample was obtained using Applied Biosystems 7300 System SDS version 1.4 software (Applied Biosystems). Relative quantitative levels were calculated on the basis of the CT value by a standard curve method and were normalized to β-actin (*ACTB*) expression (Hs99999903_m1). The experiments were done in triplicate for each sample.

### Statistical analysis

Differences in allele and genotype frequencies between SLE patients and healthy controls were analyzed by using a χ^2 ^test with 2 × 2 contingency tables. When one or more of the variables in the contingency tables was 20 or less, Fisher's exact test was employed. Linkage disequilibrium (LD) was analyzed using HaploView version 4.0 software (Broad Institute, Cambridge, MA, USA). Pairwise *r*^2 ^values were calculated on the basis of the genotypes of 274 healthy controls. Estimation of haplotype frequencies and association tests were performed using HaploView version 4.0 software.

To examine whether each SNP independently contributes to susceptibility to SLE, conditional logistic regression analysis was employed. Dominant, codominant and recessive models were tested for each SNP, and the model that provided the lowest *P *value was selected as the best fit model. As a result, the following were used as independent variables: rs3853839, C/C = 0, G/C = 1 and G/G = 2 under the codominant model for the G allele; rs179019, C/C = 0, C/A = 0, A/A = 1 under the recessive model for the A allele; rs179010, C/C = 0, C/T = 0, and T/T = 1 under the recessive model for the T allele.

The association of *TLR7 *SNPs with *TLR7 *mRNA expression was assessed by using the Kruskal-Wallis test.

## Results

### Association of *TLR7 *SNPs with SLE in a Japanese female population

To systematically examine association of *TLR7 *SNPs with SLE in Japanese, eight tag SNPs in the *TLR7 *gene, including rs3853839 in the 3'UTR, which was recently shown to be associated with SLE in East Asian populations [7], were analyzed in 344 Japanese females with SLE and 274 healthy female controls. Because *TLR7 *is located on an X chromosome, male and female individuals needed to be analyzed for the association separately. However, because of the female predominance of SLE (9:1 female to male ratio), the sample size of male SLE patients was too small to be analyzed statistically. Therefore, male patients and controls were excluded from this study. No deviation from the Hardy-Weinberg equilibrium was observed in the controls (*P *> 0.05).

**Figure 1:**
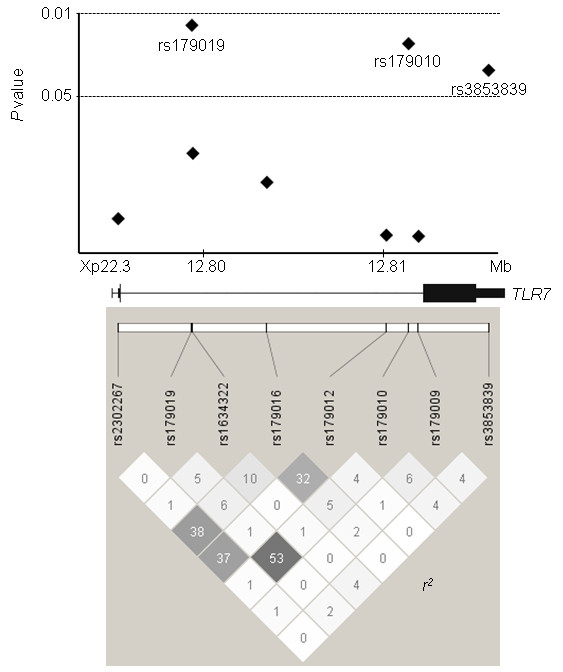
**Association of tag single-nucleotide polymorphisms in the Toll-like receptor 7gene with systemic lupus erythematosus**. Top: *P *values under the recessive model for minor alleles are indicated. Association was tested by χ^2 ^analysis using 2 × 2 contingency tables. Bottom: *r*^2 ^values based on data from 274 healthy Japanese women are shown.

In addition to the association of rs3853839 reported separately [7], the association of two SNPs in intron 2, rs179019 and rs179010, was newly detected (Figure 1 and Table 1). Significant association of rs179019 and rs179010 was observed under the recessive model for the A and T alleles, respectively (rs179019: *P *= 0.016, odds ratio (OR) 2.02, 95% confidence interval (95% CI) 1.15 to 3.54; rs179010: *P *= 0.018, OR 1.75, 95% CI 1.10 to 2.80). LD was present between rs179019 and rs179010 (*r*^2 ^= 0.53), while LD between rs3853839 and each of the intronic SNPs was modest (*r*^2 ^= 0.02 and 0.04) (Figure 1).

**Table 1:** Association of *TLR7 *SNPs with SLE in a Japanese population^a^

					Allelic association		Dominant model		Recessive model	
Study population	Genotype, *n *(%)			Risk allele, *n *(%)	*P*	OR (95% CI)	*P*	OR (95% CI)	*P*	OR (95% CI)
rs3853839	G/G	G/C	C/C	G						
SLE	197(57.3)	125 (36.3)	22 (6.4)	519 (75.4)	0.017	1.36 (1.06 to 1.75)	0.030	1.87 (1.05 to 3.31)	0.072	1.34 (0.97 to 1.84)
Controls	137(50.0)	106 (38.7)	31 (11.3)	380 (69.3)						
										
rs179019	A/A	C/A	C/C	A						
SLE	45 (13.1)	131 (38.1)	168 (48.8)	221 (32.1)	0.17	1.19 (0.93 to 1.52)	0.77	1.05 (0.76 to 1.44)	0.016^b^	2.02 (1.15 to 3.54)
Controls	19 (6.9)	118 (43.1)	137 (50.0)	156 (28.5)						
										
rs179010	T/T	C/T	C/C	T						
SLE	61 (17.7)	156 (45.3)	127 (36.9)	278 (40.4)	0.062	1.25 (0.99 to 1.57)	0.36	1.16 (0.84 to 1.61)	0.018	1.75 (1.10 to 2.80)
Controls	30 (10.9)	133 (48.5)	111 (40.5)	193 (35.2)						

^a^*TLR7*, Toll-like receptor 7 gene; SNP, single-nucleotide polymorphism; 95% CI, confidence interval; OR, odds ratio; SLE, systemic lupus erythematosus. Genotype and allele frequencies are shown in parentheses (%). Association was tested by χ^2 ^analysis or Fisher's exact test using 2 × 2 contingency tables under the indicated models for rs3853839G, rs179019A and rs179010 T alleles. ^b^Fisher's exact test was used.

To examine the contribution of each SNP to susceptibility to SLE, conditional logistic regression analysis was conducted. As shown in Table 2, the association of rs3853839 remained significant after adjustment for the intronic SNP genotypes. Adjusted *P *values (*P*_adjusted_) for rs3853839 under the codominant model were 0.040 and 0.047 after adjustment for rs179019 and rs179010, respectively. The association of rs179019 and rs179010 also remained significant after adjustment for rs3853839 (rs179019: *P*_adjusted _= 0.026; rs179010: *P*_adjusted _= 0.042). These results suggest that rs3853839 and the intronic SNPs are independently associated with SLE. In contrast, the association of rs179019 and rs179010 was eliminated when they were adjusted for each other as expected on the basis of LD between the two (Table 2 and Figure 1).

**Table 2:** Conditional logistic regression analysis of *TLR*7 SNPs^a^

				*P*_adjusted_^b^		
SNP	Risk allele	Model	*P* ^c^	rs3853839	rs179019	rs179010
rs3853839	G	Codominant	0.021	NA	0.040	0.047
rs179019	A	Recessive	0.014	0.026	NA	0.24
rs179010	T	Recessive	0.019	0.042	0.42	NA

^a^*TLR7*, Toll-like receptor 7 gene; SNP, single-nucleotide polymorphism; NA, not applicable; ^b^*P *value adjusted for each SNP by conditional logistic regression analysis using the indicated model; ^c^*P *value for each SNP calculated by logistic regression analysis. The indicated model showed the lowest *P *value for each SNP.

In agreement with these findings, when only the patients and controls carrying the risk genotypes of the 3' UTR SNP were analyzed, possession of both of the intronic SNP risk genotypes was significantly associated with SLE (*P *= 0.0043, OR 2.45, 95% CI 1.31 to 4.60) (Table 3).

**Table 3:** Independent effect of intron 2 SNPs in the carriers of the 3' UTR risk genotypes^a^

Risk genotype			Study group, *n *(%)				
rs3853839	rs179019	rs179010	SLE (*N *= 322)	Controls (*N *= 243)	*P*	OR	95% CI
G/G or G/C	A/A	T/T	42 (13.0)	14 (5.8)			
+	+	+	280 (87.0)	229 (94.2)	0.0043	2.45	1.31 to 4.60
+	Others					Reference	

^a^SNP, single-nucleotide polymorphism; 3' UTR, 3' untranslated region; OR, odds ratio; 95% CI, 95% confidence interval. Genotype frequencies are shown in parentheses (%). *P *value was calculated using Fisher's exact test.

SLE-associated SNPs rs179019, rs179010 and rs3853839 were estimated to form five major haplotypes (Table 4). When haplotype frequencies were compared between female SLE patients and healthy controls, tendencies for an increase of haplotype 3 containing all of the SLE risk alleles and a decrease of haplotype 2 containing none of them were observed, although the differences did not reach statistical significance (permutation *P*, haplotype 3 = 0.081; permutation *P*, haplotype 2 = 0.068). We next examined the haplotype association under the recessive model. Individuals homozygous for all three SNPs were considered to be homozygous for the haplotype. A significant association of haplotype 3 was detected under the recessive model (haplotype 3/3 versus others: *P *= 0.016, OR 2.37, 95% CI 1.17 to 4.80), but haplotype 1 (*P *= 0.21, OR 1.32, 95% CI 0.86 to 2.05) and haplotype 4 (*P *= 1.0, OR 0.80, 95% CI 0.11 to 5.68), which also contained the 3'UTR risk allele but not both of the intronic SNPs, were not associated. These results suggest that the combination of the intronic and 3'UTR risk alleles may be associated with higher SLE risk.

**Table 4:** Estimated haplotype frequencies in SLE and controls^a^

Haplotype	rs179019	rs179010	rs3853839	SLE	Controls	Permutation *P *value
1	C	C	G	40.6%	38.0%	0.94
2	C	C	C	18.2%	24.1%	0.068
3^b^	A	T	G	26.1%	20.3%	0.081
4	C	T	G	8.5%	8.8%	1.0
5	A	T	C	5.2%	5.6%	1.0

^a^SLE, systemic lupus erythematosus; *P *values were calculated by permutation test (100,000 permutations) using HaploView version 4.0 software; ^b^each haplotype was also tested for association under the recessive model. Individuals homozygous at all three SNPs were considered homozygous for the haplotype. Only haplotype 3 was significantly associated with SLE under the recessive model (SLE, 31 (9.0%) of 344; control, 11 (4.0%) of 274; *P *= 0.016 by Fisher's exact test; odds ratio 2.37, 95% confidence interval 1.17 to 4.80).

### Association of *TLR7 *SNPs with clinical subsets of SLE

We examined whether *TLR7 *SNPs were associated with clinical phenotypes such as the presence of anti-Sm antibodies, anti-double-stranded DNA antibodies and renal disorder. Association was tested between SLE patients with each phenotype and healthy controls. The OR of rs179019 was slightly higher in the subset with renal disorder (*P *= 0.011, OR 2.25, 95% CI 1.21 to 4.18) than in all SLE patients (*P *= 0.016, OR 2.02, 95% CI 1.15 to 3.54) (Table 5), although no statistically significant association was observed in case-only analysis (SLE patients with renal disorder versus those without). The association of rs179019 with renal disorder remained significant after adjustment for rs3853839 on the basis of logistic regression analysis (*P*_adjusted _= 0.019, OR 2.10, 95% CI 1.13 to 3.93 under the recessive model).

**Table 5:** Association study of *TLR7 *SNPs with clinical characteristics of SLE^a^

		SLE total		Anti-Sm antibodies		Anti-dsDNA antibodies		Renal disorder	
SNP	Model	*P*	OR (95% CI)	*P*	OR (95% CI)	*P*	OR (95% CI)	*P*	OR (95% CI)
rs3853839	Allele	0.017	1.36 (1.06 to 1.75)	0.032	1.65 (1.04 to 2.62)	0.014	1.40 (1.07 to 1.84)	0.025	1.40 (1.04 to 1.89)
rs179019	Recessive	0.016^b^	2.02 (1.15 to 3.54)	1.0^b^	0.89 (0.29 to 2.73)	0.029^b^	1.93 (1.07 to 3.48)	0.011^b^	2.25 (1.21 to 4.18)
rs179010	Recessive	0.018	1.75 (1.10 to 2.80)	0.67^b^	1.16 (0.51 to 2.67)	0.030	1.72 (1.05 to 2.83)	0.042	1.73 (1.02 to 2.95)

^a^*TLR7*, Toll-like receptor 7 gene; SNP, single-nucleotide polymorphism; SLE, systemic lupus erythematosus; anti-Sm, anti-Smith; dsDNA, double-stranded DNA; OR, odds ratio, 95% CI, confidence interval; ^b^Fisher's exact test was used. Association was tested by χ^2 ^analysis or Fisher's exact test using 2 × 2 contingency tables under the indicated model for rs3853839G, rs179019A and rs179010 T allele. All SLE as well as each SLE subset were compared with healthy controls.

### Analysis of association between *TLR7 *SNPs and*TLR7 *mRNA levels

**Figure 2:**
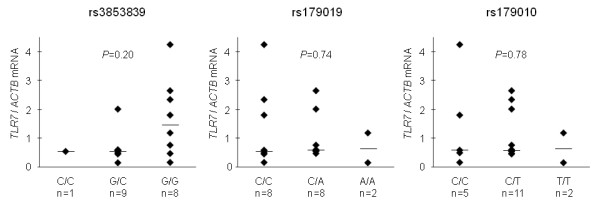
**Association analysis of *Toll-like receptor 7 *genotypes with mRNA expression in peripheral blood mononuclear cells**. Association between *Toll-like receptor 7 *(*TLR7) *single-nucleotide polymorphisms (SNPs) and *TLR7 *mRNA levels was examined by using the Kruskal-Wallis test. Relative quantitative levels of *TLR7 *mRNA were normalized to β-actin (*ACTB*) mRNA levels. Bars indicate median values in each group. The experiments were performed in triplicate.

To investigate the functional significance of the *TLR7 *SNPs, we analyzed the association between*TLR7 *SNPs and *TLR7 *mRNA levels (Figure 2). The *TLR7 *mRNA levels in PBMNCs from Japanese female SLE patients were measured using RT-PCR assay and were compared among individuals carrying each genotype. Although not statistically significant because of the limited sample size, a tendency toward an association of rs3853839G with elevated *TLR7 *mRNA levels was observed (*P *= 0.20 by Kruskal-Wallis test). This tendency was consistent with the observations in the Chinese population [7], which demonstrated increased *TLR7 *transcripts in individuals carrying rs3853839G. On the other hand, evidence for an association of the intronic SNPs with mRNA levels was not observed.

## Discussion

In the recently reported multicenter study, an association of rs3853839 was originally found by screening the *TLR7*-*TLR8 *region in Chinese and Korean populations and was subsequently replicated in Chinese and Japanese populations [7]. In the process of the study, some population difference was noted for rs3853839 and other SNPs, even among these East Asian populations. Because association between *TLR7 *and SLE had not been examined in a systematic manner in a Japanese population, we thought that *TLR7 *SNPs other than rs3853839 might also contribute to SLE.

To explore such a possibility, we analyzed the association of eight tag SNPs in *TLR7 *and the newly detected association of two SNPs in intron 2, rs179019 and rs179010. Conditional logistic regression analysis indicated that the association of the intronic SNPs cannot be explained by LD with rs3853839. In agreement with these results, the association of the intronic SNPs remained significant after excluding the effect of the 3'UTR SNP by testing the association only among individuals carrying the 3'UTR risk allele. Furthermore, haplotype analysis showed significant association of the haplotype containing all of the three SLE risk alleles, but not of the other haplotypes. All of these results support the possibility that the possession of both the 3'UTR and intronic risk alleles may confer further risk for SLE.

Although rs179019 and rs179010 were also investigated in the Discovery Panel in the previous study, the majority of whom were Chinese and Korean participants, no significant association was detected [7]. The Japanese patients and controls analyzed in this study were not included in the Discovery Panel. Population difference was also observed for rs3853839 between the Chinese and Korean populations, as this SNP was strongly associated with SLE in Chinese, but not in Koreans [7], suggesting that the genetic background with respect to *TLR7 *association with SLE might be somewhat different, even among the closely related East Asian populations. Minor allele frequencies of rs179019 and rs179010 in the HapMap CHB (Han Chinese in Beijing) samples (rs179019: 30.9%, rs179010: 37.3%) available in the International HapMap database [9] are similar to those in the Japanese observed in this study (rs179019: 28.5%, rs179010: 35.2%). Thus, the difference in the association cannot be explained by differences in the minor allele frequencies. We cannot rule out the possibility that another SNP tagged by rs179019 and rs179010 in Japanese, but not in Chinese or Koreans because of difference in the LD status, might play a causative role. Such a possibility would be addressed by resequencing the entire *TLR7 *region.

There is growing evidence to support involvement of type I IFN in the development of SLE. TLR7 is crucial for the production of type I IFN. Thus, the most plausible role of *TLR7 *SNPs in SLE pathogenesis is likely to be explained by elevated type I IFN production. The sera of SLE patients displayed elevated levels of type I IFN, and expression of IFN-inducible genes in PBMNCs was also upregulated in SLE [10]. Occasional occurrence of SLE symptoms following treatment with IFNα in patients with cancer or hepatitis underscored the relevance of type I IFN [10]. Type I IFN is thought to be a potential therapeutic target for SLE, and clinical trials of anti-IFNα antibodies in SLE are currently underway [11].

Recent genetic studies have identified an association of type I IFN pathway-related genes, IFN regulatory factor 5 (*IRF5*) and *STAT4*, with SLE in various populations [10,12-16]. An *IRF5 *SLE risk haplotype has been shown to be associated with high serum IFNα activity in SLE patients [17], whereas the *STAT4 *SLE risk variant was associated with increased sensitivity to IFNα *in vivo *[18]. These observations, as well as the previous study on *TLR7 *showing upregulation of TLR7 in the risk genotype [7], suggest that SLE-associated alleles in the type I IFN pathway are gain-of-function alleles in nature.

Another potential role of *TLR7 *polymorphisms may be related to the induction of proinflammatory cytokines. IRF5 is activated by TLR7 signaling and regulates the expression of many genes, including type I IFN and proinflammatory cytokines [19]. STAT4 is activated by type I IFN as well as interleukin 12 and plays a role in Th1 differentiation [20]. In view of these observations, the association between *TLR7 *SNPs and SLE might also be explained by overproduction of proinflammatory cytokines in addition to type I IFN.

There are conflicting reports about copy number variation (CNV) of *TLR7*. Initially, the existence of CNV was reported by Kelley *et al*. [21]. They showed that, although common CNV was observed in Caucasians and African-Americans, no association with SLE was detected [21]. Recently, García-Ortiz *et al*. [22] reported an association of CNV with childhood-onset SLE in a Mexican population. In contrast to these observations, Shen *et al*. [7] did not find common *TLR7 *CNV in multiple populations, including Asians. The latter observation is consistent with the fact that no CNV was registered in the Database of Genomic Variants [23], which includes results derived from the HapMap JPT (Japanese in Tokyo) samples.

Although our observation in the expression analysis supported the previous report that indicated the association between the risk allele of the 3'UTR SNP and elevated expression of *TLR7 *[7], evidence for the association of the intronic SNPs with levels of *TLR7 *mRNA was not observed, and therefore the molecular mechanism of the intronic SNPs requires further study. TLR7 is mainly expressed in pDCs and B cells. pDCs represent the major source of type I IFN, but constitute less than 1% of PBMNCs. If the intronic SNPs have a regulatory role in a cell type-specific fashion and influence the expression level of *TLR7 *in pDCs but not in other white blood cells, such an effect may not have been detected in the analysis of total PBMNCs. In addition, the sample size of this study may not have been large enough for us to conclude that the intronic SNPs have no effect on the expression of *TLR7*.

Because we focused only on the Japanese population, the sample size of this study was limited and the observed statistical association was modest. Therefore, the association of the intronic SNPs should be confirmed in future independent studies.

## Conclusions

*TLR7 *intronic SNPs rs179019 and rs179010 are associated with SLE independently of 3'UTR SNP rs3853839 in Japanese women. Our findings support the genetic role of *TLR7 *SNPs in Asian populations with SLE.

## Abbreviations

95% CI: 95% confidence interval; CNV: copy number variation; CpG: cytidine-phosphate-guanosine; IFN: interferon; LD: linkage disequilibrium; OR: odds ratio; PBMNCs: peripheral blood mononuclear cells; pDCs: plasmacytoid dendritic cells; RT-PCR: reverse transcription polymerase chain reaction; SLE: systemic lupus erythematosus; SNP: single-nucleotide polymorphism; ssRNA: single-stranded RNA; TLR: Toll-like receptor; UTR: untranslated region; Yaa: Y chromosome-linked autoimmune accelerator.

## Competing interests

The authors declare that they have no competing interests.

## Authors' contributions

AK participated in the study design; carried out all genotyping, expression analysis and statistical analyses; and wrote the manuscript. HF, YK, SI, TH, MK, IM, ST, YT, HH and TS recruited the patients and controls and collected clinical information. NT designed and coordinated the study and helped in the manuscript preparation. All authors read and approved the final manuscript.
